# Corticosterone as a Potential Confounding Factor in Delineating Mechanisms Underlying Ketamine’s Rapid Antidepressant Actions

**DOI:** 10.3389/fphar.2020.590221

**Published:** 2020-11-30

**Authors:** Lauren Wegman-Points, Brock Pope, Allison Zobel-Mask, Lori Winter, Eric Wauson, Vanja Duric, Li-Lian Yuan

**Affiliations:** Department of Physiology and Pharmacology, Des Moines University, Des Moines, IA, United States

**Keywords:** ketamine, hydroxynorketamine, glucocorticoids, corticosterone, rapid-acting antidepressant, stress

## Abstract

Recent research into the rapid antidepressant effect of subanesthetic doses of ketamine have identified a series of relevant protein cascades activated within hours of administration. Prior to, or concurrent with, these activation cascades, ketamine treatment generates dissociative and psychotomimetic side effects along with an increase in circulating glucocorticoids. In rats, we observed an over 3-fold increase in corticosterone levels in both serum and brain tissue, within an hour of administration of low dose ketamine (10 mg/kg), but not with (2R, 6R)-hydroxynorketamine (HNK) (10 mg/kg), a ketamine metabolite shown to produce antidepressant-like action in rodents without inducing immediate side-effects. Hippocampal tissue from ketamine, but not HNK, injected animals displayed a significant increase in the expression of *sgk1*, a downstream effector of glucocorticoid receptor signaling. To examine the role conscious sensation of ketamine’s side effects plays in the release of corticosterone, we assessed serum corticosterone levels after ketamine administration while under isoflurane anesthesia. Under anesthesia, ketamine failed to increase circulating corticosterone levels relative to saline controls. Concurrent with its antidepressant effects, ketamine generates a release of glucocorticoids potentially linked to disturbing cognitive side effects and the activation of distinct molecular pathways which should be considered when attempting to delineate the molecular mechanisms of its antidepressant function.

## Introduction

Ketamine, synthesized as a phenylcyclidine (PCP) derivative over 50 years ago, has long been used as general anesthetic acting primarily through blockade of NMDA receptors in the brain (Li and Vlisides, 2016). Ketamine, at subanesthetic (0.1–0.5 mg/kg) doses, has emerged as a clinical rapid acting antidepressant (RAAD) (Krystal, et al., 1994; Berman et al., 2000). Accumulating evidence supports ketamine’s efficacy in the treatment of major depressive disorder (MDD) and other anxiety disorders with low dosage administration (Berman et al., 2000; Zarate et al., 2006; Zarate et al., 2012; Murrough et al., 2013). Ketamine not only offers quick relief from depressive symptoms and suicidal ideation within a few hours, it is also effective in a large subset of the treatment‐resistant population who do not respond to current mainstream monoamine therapies (Aleksandrova et al., 2017). Despite ketamine’s unique clinical advantages, its use as a safe, long-term treatment has limitations. Even at subanesthetic doses, ketamine produces psychotomimetic and dissociative effects within minutes of administration, increasing the potential for abuse, largely restricting administration to a clinical setting.

After administration ketamine is metabolized to a series of structurally distinct metabolites, with the potential to activate unique pathways. Intense research into the RAAD effect of subanesthetic doses of ketamine have identified a series of protein cascades and transcriptional programs activated within an hour of administration and associated with increased neuronal connectivity and excitability (Zanos and Gould 2018). Among the multiple mechanisms related to ketamine’s rapid action, are rapid enhancement of glutamate transmission and production of BDNF in the hippocampus, activation of mTOR signaling pathways in the prefrontal cortex (PFC) and suppression of neuronal firing in lateral habenula (Garcia et al., 2008; Li et al., 2010; Autry et al., 2011; Duman et al., 2012; Yang et al., 2018). Recent evidence in rodents highlights the potential contributions of a ketamine metabolite (2R, 6R)-hydroxynorketamine (HNK), indicating HNK is sufficient to reproduce the antidepressant effects seen with ketamine, including increased neuroexcitability and synaptic strengthening (Zanos et al., 2016). HNK is purported to be unique in its NMDAR‐independent activity and its apparent lack of dissociative effects which appear to manifest as locomotor deficits in rodent models (Danysz et al., 1994). However, consensus in the contributions of various metabolites and their neural mechanisms has yet to be developed and major questions remain, including whether ketamine’s antidepressant action is NMDAR-dependent or independent (Aleksandrova et al., 2017; Zanos and Gould 2018).

Since the discovery of ketamine’s antidepressant effects there has been a flood of research trying to determine the mechanisms behind its rapid action and unprecedented success in treatment‐resistant populations. There has also been an effort to uncouple its antidepressant mechanisms from its psychotropic effects which would allow more flexibility in clinical use. The ketamine metabolite HNK, with its intriguing antidepressant-like effects in animal models and lack of outward side effects, seemed to be an ideal candidate to help tease apart ketamine’s antidepressant mechanisms from the undesirable side effects. We sought to compare the downstream molecular pathways of ketamine and HNK administration, note any differences, and begin to identify pathways unique to the dissociative effects of ketamine. Our results did reveal molecular differences on an unexpected platform, namely activation of the hypothalamic-pituitary-adrenal (HPA) axis. HPA axis hyperactivity, along with the generation of glucocorticoid resistance, has repeatedly been linked to the manifestation of MDD making effects of ketamine on this pathway of particular interest when parsing out its RAAD mechanisms (Pariante 2009). This report will further characterize this phenomenon of ketamine administration versus HNK, including gender differences, and begin to address the mechanisms of its generation.

## Materials and Methods

### Animals

Sprague-Dawley male and female rats between the ages of 8–10 weeks old were obtained from Charles River (Wilmington, MA). Male and female rat cohorts were done separately. The animals were pair-housed under a 12-h light/dark cycle (lights on at 6:00 am) with controlled temperatures and humidity. Food and water were administered ad libitum. Body weight was recorded on a weekly basis, and overnight fluid consumption was monitored twice per week. The use of animals for these studies was approved by the Des Moines University Institutional Animal Care and Use Committee.

### Drugs

(2R,6R)-hydroxynorketamine hydrochloride (R&D Systems, Minneapolis, MN) was dissolved in saline at 10 mg/ml immediately prior to use. Prior to administration, Ketamine hydrochloride (Ketaset, Zoetis, US) was diluted from 100 mg/ml to 10 mg/ml in saline. Corticosterone hemisuccinate (4-pregen-11B 21-DIOL-3 20-DIONE 21 hemisuccinate, MW 446.53) (Steraloids, Newport RI) was added to tap water at concentration of 64.4 mg/L (producing a final concentration of 50 μg/ml corticosterone), brought to pH 12.5 using NaOH, and allowed to dissolve overnight at 4°C on a stir plate. The pH was adjusted to between 7.2 and 7.5 with HCl before administration to the animals. 25 and 12 μg/ml doses were prepared in the same manner with 32.2 and 16.1 mg/L of corticosterone hemisuccinate respectively.

### Chemically‐Induced Stress Model

**FIGURE 1:**
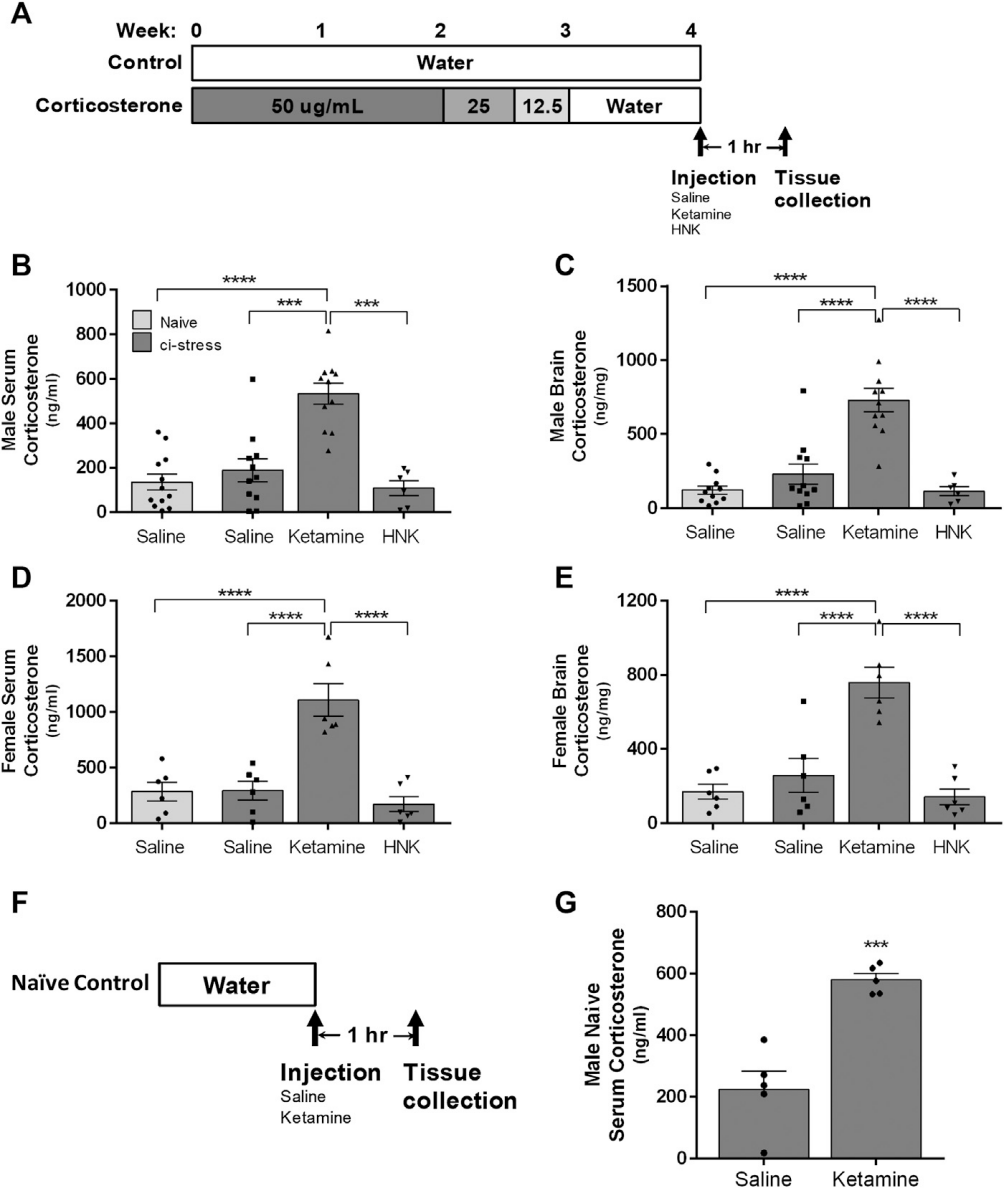
Schematic representation of the timeline for oral corticosterone exposure, drug administration, and tissue collection*.* **(A)** The rats were provided with water containing 50 μg/ml of corticosterone for 2 weeks, after which the dosage was tapered off with 3 days to 25 μg/ml followed by three more days at 12.5 μg/ml. The rats were returned to regular drinking water for 3–7 days to wash out exogenous corticosterone before injection and sacrifice. The rats with access to drinking water throughout the 4-week period served as naïve controls. *Measurement of corticosterone levels in serum and brain tissue in male and female rats 1-h post injection.* Ci-stress male and female rats received injection of saline (male n = 11; female n = 6), ketamine (10 mg/kg) (male *n* = 11; female *n* = 6) or HNK (10 mg/kg) (male *n* = 6; female *n* = 6). Naïve rats received only saline (male *n* = 12; female *n* = 6). **(B)** Circulating corticosterone levels in male rats. **(C)** Cortical brain levels of corticosterone in male rats. **(D)** Circulating corticosterone levels in female rats. **(E)** Cortical brain levels of corticosterone in female rats. *Measurement of serum corticosterone levels 1-h post injection of naïve rats* **(F)**. Timeline of naïve male treatment and tissue collection. **(G)** Ketamine injection caused an increase in circulating corticosterone versus saline injection in naïve rats (saline *n* = 5, ketamine *n* = 5). [(**B–D)** ***p < 0.001, ****p < 0.0001, two-way ANOVA with Tukey post-test, (**G)** ***p < 0.001, student t-test).

Corticosterone was administered to the rats through their drinking water according to Gourley et al. (Gourley, Wu et al., 2008; Gourley and Taylor 2009). The rats were provided with water containing 50 μg/ml of corticosterone for 2 weeks. A freshly prepared solution was provided every 3 days. Daily dosages corticosterone of were calculated using overnight water consumption values and body weight. 5–7 mg/kg was considered the minimum effective dosage. After the initial 2 weeks, the dosage was tapered off with 3 days at 25 μg/ml followed by three more days at 12.5 μg/ml. The rats were returned to regular drinking water for 3–7 days to wash out exogenous corticosterone before injection and sacrifice (Figure 1A). The rats with access to drinking water throughout the 4-week period served as naïve controls.

### Blood and Tissue Collection

Animals were euthanized by decapitation 1-h after receiving an intraperitoneal (i.p.) injection of saline, ketamine or HNK. Each treatment group contained five to six animals. Two male cohorts and one female cohort were used. Treatment groups were staggered throughout the day to avoid natural oscillations in circadian pathways from affecting any one treatment group. Trunk blood was collected in serum collection tubes (BD Bioscience) and allowed to clot 15–30 min before centrifugation. Brain tissue was dissected, snap frozen using liquid nitrogen, and stored at −80°C until use.

### Blood Collections Under Anesthesia

8 to 12 week old male animals were sedated using 3% isoflurane in a ventilated chamber. After initial sedation, the animals were maintained under anesthesia at 1.5% isoflurane through a nosecone. A heating pad was used to maintain body temperature. Hind limbs were shaved and treated with a chemical depilatory to allow visualization of the saphenous vein. A 23.5-gauge needle was used to puncture the saphenous vein and blood was collected using a serum capillary blood collection tube (Microvette 200, Sarsedt, Germany). After the initial blood collection, an i.p. injection of saline (*n* = 7) or ketamine (10 mg/kg) (*n* = 7) was administered. The animals remained under anesthesia for 60 min after the injection when blood was collected again through the saphenous vein. Animals were allowed to recover and returned to their cages.

### Measurement of Serum and Brain Tissue Corticosterone Levels

For measurement of endogenous corticosterone levels in serum and cortical brain tissue, we utilized the DetectX CORT ELISA kit (Arbor Assays, Ann Arbor, MI). For the cortical tissue, a separate steroid ethyl acetate extraction procedure was required before corticosterone measurement. The assay was performed according to the manufacturer’s instructions, which each sample run in duplicate. Samples were compared to a standard curve using a 4-parameter logistical regression model.

### RNA Extraction and qPCR

Total RNA was extracted from the entire hippocampus using the RNeasy Lipid Tissue Mini kit (Qiagen, Germantown, MD). cDNA was generated using the High Capacity cDNA Reverse Transcription Kit (Applied Biosystems, Waltham, MA) using random hexamer primers. qRT-PCR was performed on a CFX Connect Real Time PCR Detection System (Bio-Rad, Hercules, CA) utilizing iTaq Universal SYBR Green Supermix (Bio-Rad, Hercules, CA). B-Actin and GAPDH were both used as control genes in all experiments. Fold changes were calculated using the ΔΔCt method. B-Actin: (PrimeTime® IDT, Coralville, IA) Forward 5ʹ-TCA​CTA​TCG​GCA​ATG​AGC​G-3ʹ, Reverse 5ʹ-GGC​ATA​GAG​GTC​TTT​ACG​GAT​G-3ʹ. Primer 3 software was utilized to design SGK-1 and GAPDH primer sets. SGK-1 Forward 5ʹ-GTG​CCT​TGC​TAG​AAG​AAC​CTT​TCC-3ʹ, Reverse 5ʹ-CTC​ACC​TCC​TCC​AAG​TCC​CTC​TC-3ʹ. GAPDH Forward 5ʹ-GTC​ATG​AGC​CCT​TCC​ACG​ATG​C-3ʹ, Reverse 5ʹ-ACA​ACT​CCC​TCA​AGA​TTG​TCA​GC-3ʹ (Koressaar and Remm 2007; Untergasser et al., 2012).

### Statistics

Two-way ANOVA was performed to compare the effects of gender and treatment condition on corticosterone levels. The 1-h timepoint qPCR results were also analyzed by a two-way ANOVA comparing gender and treatment effects using the ΔCt values. Tukey multiple comparison tests were conducted to examine significant effects between treatment groups. Eta-squared was used to calculate all effect sizes. An unpaired student’s t-test was utilized in comparison of corticosterone levels in all naïve and 24-h animal experiments. Graphpad Prism was used to perform all statistical analyses.

## Results

### Increase in Circulating Corticosterone With Ketamine Treatment

In order to sensitize the CNS to ketamine and HNK treatment and amplify potential molecular differences we utilized a **c**hemically‐**i**nduced chronic **stress** (ci-stress) model where rats were orally exposed to exogenous corticosterone for an extended period of time (Wellman 2001; Gourley et al., 2008; Gourley and Taylor 2009; Joffe et al., 2020). The experimental timeline, which includes a weaning process to facilitate the recovery of endogenous corticosterone production, is shown in Figure 1A. This protocol is effective in inducing pro-depressive like behaviors including anhedonia, anxiety, and helplessness (Gourley et al., 2008).

One hour prior to euthanasia animals were injected with saline, ketamine (10 mg/kg) or HNK (10 mg/kg). We chose this timepoint because protein cascades initiated within the first hour of ketamine or HNK administration are critical to its antidepressant effect (Li et al., 2010; Zanos and Gould 2018). 10 mg/kg represents a subanesthetic dosage that has consistently been shown to produce RAAD effects in rodents (Li et al., 2010; Zanos et al., 2016; Jiang et al., 2017). ELISA analysis of trunk blood indicated a substantial increase in circulating endogenous corticosterone only in the animals treated with ketamine (F (3,55) = 37.53, *p* < 0.0001, η^2^ = 0.62) (Table 1). Ketamine-injected male rats showed several folds more serum corticosterone than naïve animals injected with saline (*p* < 0.0001) and the remaining ci-stress treatment groups, treated with either saline (*p* < 0.001) or HNK (*p* < 0.001) (Figure 1B; Table 1).

**TABLE 1:** Increased levels of circulating corticosterone in the blood correlates with increased concentrations found in tissue from the cerebral cortex.

Gender	Treatment	Blood (ng/ml)	Brain (ng/mg)
Male	Naïve Saline	136.1 ± 35.6	120.9 ± 26.74
	ci-stress-saline	189.4 ± 51.6	229.6 ± 68.12
	ci-stress-ketamine	532.8 ± 47.3****	729.9 ± 78.75****
	ci-stress-HNK	108.7 ± 33.3	113.5 ± 30.0
Female	Naïve Saline	284.2 ± 84.3	169.2 ± 40.57
	ci-stress-saline	292.2 ± 83.2	257.8 ± 92.0
	ci-stress-ketamine	1106 ± 146.0****	758.0 ± 81.46****
	ci-stress-HNK	170.3 ± 68.2	141.2 ± 43.2

**** *p* < 0.001 two-way ANOVA.

Females exhibited the same pattern as the males in response to ketamine treatment, however their corticosterone response exceeded that of the males. Ketamine injected ci-stress females displayed serum corticosterone levels almost four times higher than naïve saline-injected animals and the other ci-stress groups (*p* < 0.0001) (Figure 1D; Table 1). The gender effect is moderate but significant (F (1,56) = 20.64, *p* < 0.0001, η^2^ = 0.10). The interaction effect between treatment and gender was also significant, (F (3,56) = 6.095, *p* = 0.0012, η^2^ = 0.09). These surges in serum corticosterone seen in both male and female rats were well above the daily rise in corticosterone levels seen due to normal circadian oscillations.

In order examine the possibility the observed increases in circulating endogenous corticosterone were an artifact of our ci-stress treatment protocol, we also determined the effect of ketamine on corticosterone levels in naïve animals. Male animals were given an i.p. injection of saline or ketamine and euthanized by decapitation at 1-h to collect blood and tissue (Figure 1F). Serum corticosterone levels were significantly elevated in the naïve animals treated with ketamine versus those receiving saline (*p* < 0.0005) (Figure 1G). These results indicate that ketamine induced an acute corticosterone release regardless of whether the animals were naïve or chemically stressed.

### Corticosterone Increase in Brain Tissue

Next we examined corticosterone levels in cortical brain tissue collected at the 1-h post-injection timepoint. Corticosterone, as a steroid hormone, can readily pass through the blood-brain barrier and cell membranes, therefore analysis of a single brain region should be representative of all regions (de Kloet et al., 2009). We chose the cerebral cortex to illustrate corticosterone levels throughout the CNS, thereby preserving the nuclei more relevant to depression and anxiety for a more detailed molecular analysis. Steroid extracted cerebral cortical tissue was analyzed by ELISA for corticosterone levels. Again, we saw a significant increase in corticosterone levels in the brain tissue from both male and female ketamine-treated ci-stress animals (F (3,55) = 37.53, *p* < 0.0001, η^2^ = 0.63). Male and female ketamine-ci-stress animals showed drastically higher corticosterone levels versus naïve saline-injected and the other ci-stress groups treated with saline or HNK (*p* < 0.0001) (Figure 1C and 1E; Table 1). Interestingly, unlike in the circulating corticosterone levels, there was no significant gender main effect (F (1,55) = 0.4684, *p* = 0.4966, η^2^ = 0.0012).

### Analysis of Corticosterone-Regulated Transcripts

**FIGURE 2:**
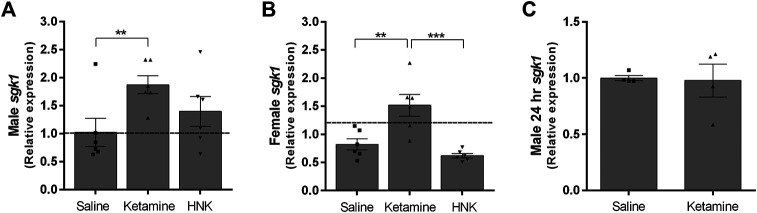
Expression of glucocorticoid effector sgk1 gene expression in male and female rats 1-h or 24-h post injection expressed as fold change versus naïve controls*.* **(A)** *Sgk1* expression in HPC of ci-stress male rats versus controls 1-h after injection (*n* = 6 for each condition). **(B)** *Sgk1* expression in HPC of ci-stress female rats versus controls 1-h after injection (n = 6 for each condition). **(C)** *Sgk1* expression in HPC of male rats 24 h after injection (n = 4 for each condition). All values are presented as a fold change from stress control animals injected with saline. (**p < 0.01, ***p < 0.001 two-way ANOVA with Tukey post-test on ΔCt values).

Glucocorticoids bind to intracellular receptors which lead to the modification of various transcriptional programs (Madalena and Lerch 2017). Serum glucocorticoid kinase 1 (*sgk1*) is a well-documented downstream effector of glucocorticoid signaling involved in regulating ion channel activity and neuronal excitability (Lang et al., 2010). We examined *sgk1* expression as a gauge of increased glucocorticoid signaling within the hippocampus. We chose the hippocampus for our analysis, because as part of the limbic system, it is integrally involved in the animal’s stress response. Ketamine treatment caused a significant increase in *sgk1* expression in both genders (F (3,40) = 11.20, *p* < 0.0001, η^2^ = 0.4). While the gender effect was not significant, it did exert a small but significant modulating effect on treatment (F (3,40) = 3.093, *p* = 0.0376, η^2^ = 0.11). Male ketamine-injected ci-stress animals displayed a significant almost 2-fold increase in *sgk1* transcripts over both naïve and ci-stress animals injected with saline (*p* < 0.001) (Figure 2A). Ketamine-injected ci-stress females had significantly higher *sgk1* expression levels compared to both saline (*p* < 0.01) and HNK-injected (*p* < 0.001) ci-stress animals (Figure 2B). We also examined *sgk1* mRNA levels in a subset of ci-stress male rats at 24‐h post-injection to see if the levels had returned to baseline. 24‐h after treatment with either saline or ketamine, hippocampal *sgk1* mRNA levels were indistinguishable between groups (Figure 2C). Both male and female 1-h treatment groups and the male 24-h treatment groups were also examined for differences in FKBP5 expression, another glucocorticoid receptor downstream target, however there was no change in expression at either the 1- or 24-h timepoints for any of the groups (data not shown). This was likely due to the timing of the tissue collection, and additional timepoints would allow for a more thorough characterization of corticosterone’s downstream effects.

### Ketamine Effects on Circulating Corticosterone Under Anesthesia

**FIGURE 3:**
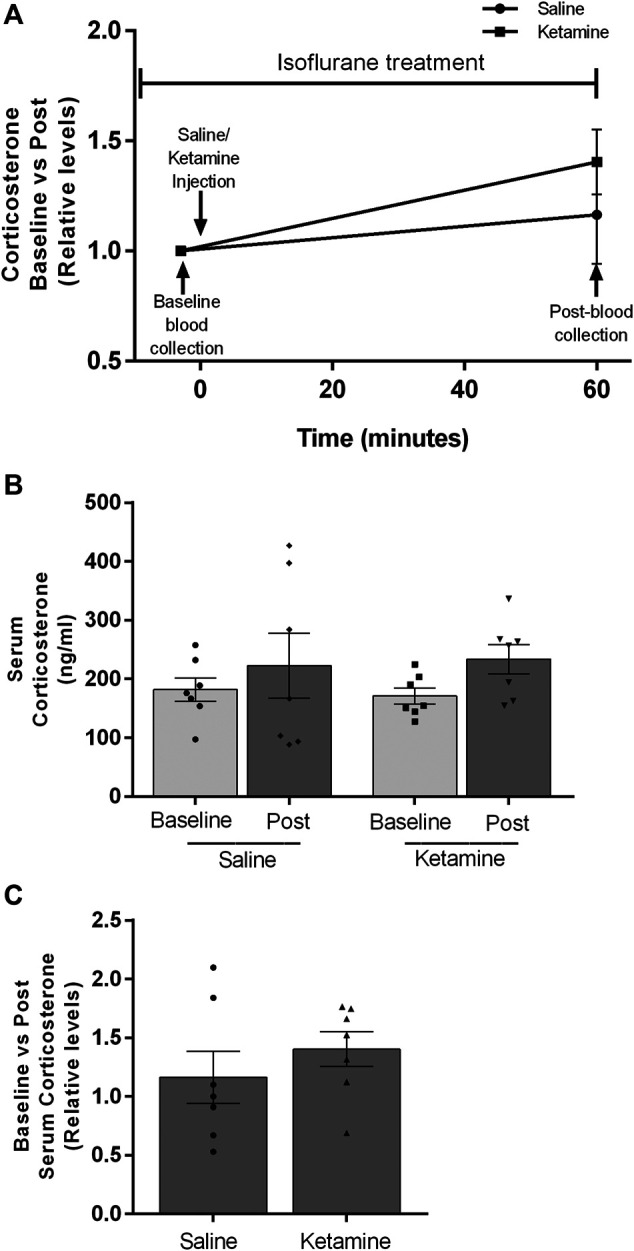
*Circulating corticosterone levels of male rats under isoflurane anesthesia before and after saline or ketamine injection.* **(A)** Timeline showing the experimental design. Blood collection and injection timepoints are noted with black arrows. **(B)** Post-injection corticosterone levels for saline and ketamine (*n* = 7 for both groups). **(C)** Fold increases between pre and post corticosterone levels after injection.

Within minutes of administration of subanesthetic doses of ketamine, freely mobile conscious rats begin to exhibit locomotor deficits including ataxia and stereotypic behavior (Danysz et al., 1994; Razoux et al., 2007; Zoupa et al., 2019). This is embodied by a staggered gait, weaving and falling over. The severity of the locomotor effect has a positive correlation with increasing ketamine dosages (Imre et al., 2006). We hypothesized this behavior is an outward manifestation of the dissociative effects described by human subjects in response to subanesthetic ketamine administration. We reasoned this novel experience could generate confusion and anxiety in the animal resulting in activation of the HPA axis. To determine whether the observed ketamine-induced corticosterone release is linked to the animal’s conscious perception of the drug’s effects, we examined its impact on corticosterone levels under anesthesia. We anesthetized naïve male rats with isoflurane. Isoflurane was chosen based on research showing it to have minimal effect on circulating corticosterone levels in male rats (Wu et al., 2015; Bekhbat et al., 2016). Importantly, isoflurane also appears to leave the HPA axis intact as repeated blood draws under anesthesia increase circulating corticosteroids in humans, rodents and rabbits (Vachon and Moreau 2001; Nishiyama et al., 2005; Gil et al., 2007). Our experimental design is depicted in Figure 3A. There was no statistical difference between anesthetized saline and ketamine groups in pre-injection corticosterone levels (181.9 ± 19.8 vs 170.9 ± 13.45, *p* = 0.6541), or in the post-injection corticosterone levels (222.9 ± 55.2 vs 233.8 ± 24.9, *p* = 0.8595) (Figure 3B). There was a modest upwards trend in the ketamine pre versus post corticosterone levels (Mean of Diff 62.87 ± 26.97, *p* = 0.056), however this trend did not reach significance. Importantly, the fold changes seen in the ketamine group did not approach the levels seen in conscious animals (Figure 3C).

## Discussion

Glucocorticoids mediate the body’s response to stressful situations. Cortisol, corticosterone in rodents, is released from the adrenal cortex in response to pathways initiated in the hypothalamus and pituitary gland. Research has shown that depression and the HPA axis are intimately intertwined (Pariante and Miller, 2001). In fact, several studies document an increase in volume in both the pituitary and adrenal glands in patients with MDD (Pariante, 2009). Glucocorticoid signaling in acute bouts, has physiological properties and contributes to learning and coping skills (Cattaneo and Riva, 2016). However, when the signaling persists, due to prolonged stress or when coping is not accomplished, the effects of glucocorticoids become pathological (Raison and Miller, 2003).

Because of the pathological effects of continued exposure to glucocorticoids, prolonged treatment with corticosterone has been utilized as a model for stress-induced depressive behaviors in rodents. While not able to recapitulate all the complexities of MDD, this model can be a useful tool to sensitize the system to antidepressant treatments allowing the unveiling of slight molecular differences not detectable in naïve animals. In this vein, we used a chronic oral corticosterone treatment protocol preceding experimental or saline injections to identify differences between ketamine and HNK treatment. Following the wash-out period of the chemically-induced stress protocol, the HPA axis activity returns to baseline. Corticosterone measurements from serum and brain tissue presented here represent the endogenous levels as exogenously administered corticosterone has been cleared out of the body system. We observed that ketamine generated an acute, robust, several-fold increase in serum corticosterone versus controls, however there was no such effect in animals treated with HNK or saline. Gender played a role in the effect of ketamine administration on circulating corticosterone, with females displaying significantly increased levels compared to males. This difference is not unprecedented with females showing increased HPA activation in response to novel and acute stressors (Lesniewska et al., 1990; Weinstock et al., 1998). Additionally, female rats have been shown to display a larger motor disruption than age matched males, which could also account for the increased stress response (McDougall et al., 2017). However another independent study reported no gender difference on locomotor activity in rodents (Zanos et al., 2016).

While the activation of the HPA axis, and resultant glucocorticoid release, in response to ketamine administration has been reported (Nisticò et al., 1978; Krystal et al., 1994; Radford et al., 2018), our studies reveal additional information on sex differences, absence with HNK treatment and the potential link to dissociative/locomotor side effects. The timing of this glucocorticoid release, peaking within 30–120 min, depending on route of administration, in rodent models and human subjects, is within the activation window of protein cascades critical for ketamine’s RAAD activity, and the initiation of ketamine’s therapeutic effects which occur within 2-h of administration (Krystal et al., 1994). The release is transient, with levels returning to baseline within a couple hours of treatment completion, in both rodents and humans (Krystal et al., 1994; Khalili-Mahani et al., 2015; Radford et al., 2018). The timing of this release, exclusiveness to ketamine administration and the documented role of the HPA axis in depression all make further characterization of this phenomenon relevant to the determination of ketamine’s antidepressant mechanism.

As a consequence of its global permeation, local responses to glucocorticoids in the CNS depend on the differential expression of intracellular receptors, namely the mineralocorticoid and glucocorticoid receptors, with the hippocampus CA1 region having the highest concentration of glucocorticoid receptors (GR) within the CNS (Pariante and Miller, 2001; Madalena and Lerch, 2017; Joels, 2018). Upon glucocorticoid binding, the GR complex interacts with genes containing a Glucocorticoid Response Element (GRE) in their promoter region (de Kloet et al., 2009). By analyzing levels of *sgk1* mRNA, a GRE containing gene, in hippocampal tissue from our animals, we were able to confirm that the GR pathway is active in a nucleus shown to be intimately involved in the manifestation of depression and anxiety.

Clinical and postmortem studies of MDD patients show the hippocampus, part of the limbic system and the primary stress response circuit, has evidence of atrophy, reduced neurogenesis and an overall reduction in volume (Duric and Duman 2013; Cattaneo and Riva 2016; Boku et al., 2018). Because of its role in depression, much of the research into ketamine administration has focused on the hippocampus and its reciprocal connections with the prefrontal cortex (Carreno et al., 2016). Research into ketamine’s RAAD effects has concentrated on the excitability of hippocampal tissue and analysis of the dendritic spine number and morphology, both of which are acutely affected by downstream activity of the GR (Yuen et al., 2011; Widman and McMahon, 2018). In fact, it has been shown that a single 1-h acute stressor is sufficient to completely alter the dendritic spine landscape of the hippocampus (Sebastian et al., 2013).

How does a canonical NMDAR antagonist exert effects on corticosterone release? There are potential direct biochemical mechanisms that could generate the observed increase in corticosterone levels in our ketamine-treated animals. Sympathomimetic properties acting on the adrenal gland or direct binding to the pool of NMDARs in the hypothalamus (Radford et al., 2018). However, we contend that it is not a direct biochemical effect of ketamine binding. We suggest it is part of an acute behavioral stress response to the experience of ketamine’s dissociative effects. In rodents, ketamine generates a lack of motor coordination within minutes after injection which we believe represents the dissociative effects of ketamine reported in humans. We contend this novel and potentially stressful experience causes activation of the HPA axis and the acute corticosterone release we observed. Our removal of the animal’s conscious experience of ketamine’s effects through anesthesia, prevented an increase in circulating corticosterone. While we did see a slight non-significant rise in corticosterone levels, this was likely due to the physical trauma of repeated blood draws and did not approach the levels seen in conscious animals. Due to the number of animals involved in each cohort, we only examined a single subanesthetic ketamine dose. However, it would be of interest to see if the positive correlation between subanesthetic ketamine dosage and locomotor deficits translates into increasing levels of circulating corticosterone (Imre et al., 2006). While not definitive, it would lend further evidence to our hypothesis that this is an acute stress response to the disruption of the animal’s motor coordination.

The concurrent increase in corticosterone with ketamine administration raises the question whether the activation of the HPA axis and its downstream pathways are a necessary component to its antidepressant effect. Here we show increased HPA axis activation in females versus males in response to ketamine treatment, similarly females have been shown to have a more robust RAAD response to ketamine administration than males (Carrier and Kabbaj, 2013; Zanos et al., 2016). Could HPA axis activation contribute to ketamine’s RAAD effects and explain some of the sex differences in its effectiveness? While warranting additional investigation, several human studies have shown a link between the severity of the dissociative effects and the strength of the antidepressant effect (Zarate et al., 2006; Luckenbaugh et al., 2014; Niciu et al., 2018). Interestingly, the antidepressant effect is not seen with anesthetic doses of ketamine in mice (Li et al., 2010). Conversely, HNK does not cause locomotor defects, nor does it increase circulating corticosterone levels, yet Zanos et al. still reported RAAD effects using this compound. However, as stated previously, the assertion that HNK is sufficient to generate RAAD effects has recently come under scrutiny (Yamaguchi et al., 2018). Future experiments to see if animals treated with subanesthetic doses of ketamine under anesthesia experience a sustained antidepressant effect at 24-h might help answer the question of whether dissociation is relevant to ketamine’s RAAD effects.

Uncovering the salient pathway(s) leading to ketamine’s sustained antidepressant effects could possibly lead to the generation of treatments with the same RAAD effects while avoiding the undesirable and abuse-related side effects. However, there remains the possibility that the sustained antidepressant effects cannot be uncoupled from the dissociative effects. Could these pathways converge, ultimately working in synchrony to increase synaptic strengthening and the sustained relief of depressive symptoms? At the very least, because glucocorticoid signaling is implicated in so many pathways relevant to a depressive phenotype, the surge in circulating glucocorticoids should be considered when analyzing any results from ketamine administration. Here we have shown that outside of ketamine’s documented effects on glutamate signaling, there is a secondary behavioral component that has molecular consequences. The conscious perception of ketamine’s dissociative effects (locomotor deficits in rodents) appears to initiate its own set of secondary outcomes adjacent to the NMDAR antagonist pathway. With such a thrust in ketamine research, and no definitive answers at hand, it is becoming increasingly apparent that we must consider that ketamine’s antidepressant effect may lie in a distinct combination of events instead of a single receptor target.

## Data Availability Statement

The raw data supporting the conclusions of this article will be made available by the authors, without undue reservation.

## Ethics Statement

The animal study was reviewed and approved by Institutional Animal Care and Use Committee (Des Moines University).

## Author Contributions

Research design: LW-P, EW, VD, and LY. Conducted experiments: LW-P, BP, AZ-M, LW, VD, and LY. Data analysis: LW-P, EW, VD, and LY. Figure design and editing LW-P, AZ-M, LY. Manuscript preparation and editing: LW-P, VD, EW, and LY. All authors have given final approval of the version to be published.

## Funding

This work was supported by National Institutes of Health grants (MH115396 and MH108043 to LY).

## Conflict of Interest

The authors declare that the research was conducted in the absence of any commercial or financial relationships that could be construed as a potential conflict of interest.
